# LINE-1 Retroelements Get ZAPped!

**DOI:** 10.1371/journal.pgen.1005364

**Published:** 2015-07-16

**Authors:** Richard N. McLaughlin, Harmit S. Malik

**Affiliations:** 1 Division of Basic Sciences, Fred Hutchinson Cancer Research Center, Seattle, Washington, United States of America; 2 Howard Hughes Medical Institute, Fred Hutchinson Cancer Research Center, Seattle, Washington, United States of America; Stanford University School of Medicine, UNITED STATES

An acute viral infection begins a cascade of events in host organisms. Primed by an interferon response, host cells dramatically increase the expression of hundreds of interferon-stimulated genes (ISGs) that collectively attempt to block the virus at various stages of its intracellular cycle. The rapid kinetics and pathogen-specific action of the ISG response can dictate the outcomes of viral infections. While acute viral infections can dramatically affect the fitness of host populations, most organisms also face a fundamentally chronic conflict with the endogenous retroelements within their genomes. The replication of these genomic elements can have a profound influence on host fitness and indelibly alter host genomes. As testament to this ancient conflict, approximately 17% of the human genome is comprised of (mostly decrepit) LINE-1 elements, while at least another 30% is the direct result of LINE-1-mediated retrotransposition [1]. Nearly a hundred different human diseases have been associated with novel LINE-1 or LINE-1-facilitated germline insertions [2]. Negative consequences of retrotransposition have selected for a suite of host restriction factors, which limit the reproductive success of these elements. One major mechanism of retroelement restriction occurs via proteins that target transcriptional silencing machinery to transposons, including LINE-1 elements [3]. Recent studies have highlighted that this targeting is evolutionarily nimble and co-evolves with the elements [4]. Post-transcriptional restriction factors form a second layer of genome defense, targeting a variety of steps in the life cycle of LINE-1.

Intriguingly, some host restriction factors, such as the APOBEC3 genes [5], can do “double-duty,” acting to restrict both viruses and LINE-1 retroelements. As further demonstration of this functional duality, two complementary papers in *PLOS Genetics* by Moldovan and Moran, and Goodier et al. now demonstrate that ZAP (Zinc finger Antiviral Protein, also called ZAP-S), an ISG initially identified as an antiviral factor, also possesses LINE-1 restriction activity [6,7]. This work uncovers commonalities and some intriguing differences in ZAP’s restriction of its diverse targets.

Since LINE-1 encodes only two proteins, it relies upon protein–protein and protein–RNA interactions with multiple host proteins to complete its retrotransposition cycle. Many of these interactions represent opportune targets for host factors to curb LINE-1 retrotransposition, and they provide the basis for two complementary screens for LINE-1 restriction factors that motivate the accompanying reports.

Moldovan and Moran used immunoprecipitation and mass spectrometry to identify host factors that interact with the ORF1p RNA-binding and chaperone protein of LINE-1 [6]. Some of the interacting proteins identified were also previously identified in similar screens or were shown to play a role in LINE-1 biology [8–11]. In contrast to the agnostic survey of the first group, Goodier et al. employed a more targeted approach of testing a panel of interferon alpha-stimulated genes for their ability to restrict LINE-1 [7]. Although the relevance of the interferon response for endogenous LINE-1 restriction is unclear, the authors nevertheless find that interferon itself and a number of interferon-stimulated genes restrict LINE-1, including several proteins previously not known to restrict LINE-1, such as Mx2 and ISG20.

One gene that emerged from both screens and forms the major focus of both reports is the antiviral gene ZAP. ZAP was first identified in a screen for host antiviral factors against Moloney murine leukemia virus [12]. Subsequently, ZAP was also shown to restrict a diverse group of viruses, including retroviruses and alphaviruses [12,13]. Binding of the viral RNA by the zinc finger domain in ZAP, together with its subsequent recruitment of the exosome, specifically degraded viral transcripts [12,14]. ZAP also represses translation of viral mRNA by blocking the interaction of translation initiation factors eIF4G and eIF4A [15].

Both new reports show that human ZAP can inhibit human LINE-1 and that depletion of endogenous ZAP increases the retrotransposition of a LINE-1 reporter several fold. ZAP over-expression decreases the cellular levels of LINE-1 transcripts and proteins. This might occur in a similar fashion as ZAP’s previously described virus restriction mechanism—ZAP zinc fingers bind LINE-1 transcripts, inhibit translation, and recruit exosomes to degrade LINE-1 RNA. However, ZAP co-localization with LINE-1 ORF1 protein at cytoplasmic stress granules might suggest an alternate mode of ZAP restriction. Stress granules (SGs) are sites of storage for stalled translation complexes that likely function as regulatory compartments for translation of cellular and pathogen mRNAs; intriguingly, ZAP is a crucial component of SGs, and ZAP overexpression enhances SG formation [16]. SGs also frequently exchange mRNAs with cytoplasmic P-bodies (cytoplasmic granules containing RNA degradation proteins) [17]. Thus, it is possible that LINE-1 transcript recruitment to stress granules via ZAP leads to its eventual degradation in cytoplasmic P-bodies. Despite many attempts, the new reports are unable to firmly implicate or exclude the role of exosomes or P-bodies in ZAP restriction of LINE-1; this remains a promising avenue of future research into the mechanism of ZAP restriction. In addition to its direct effects on pathogen RNA, ZAP has been shown to dramatically increase interferon signaling via activation of the RIG-I pathway in cells [18]. Thus, some of ZAP’s anti-LINE-1 activity might be attributable to its activation of other ISGs. Investigating whether ZAP restriction of LINE-1 is independent of RIG-I might be one means to distinguish between these possibilities.

These new findings add ZAP to the growing list of restriction factors originally characterized as exogenous virus targeting factors and later shown to also restrict endogenous elements. This overlap highlights common mechanisms and features that host genomes employ to recognize foreign or endogenous invaders to curb their deleterious nature. How does ZAP distinguish self versus non-self? Many host restriction factors function by recognizing some aspect of a pathogen that differs from the other proteins and nucleic acids in the cell. For example, the cytoplasmic DNA produced from the reverse transcription of retroviral RNA or infection by a DNA virus serves as both a signal to activate restriction programs and a target for specific restriction factors [19]. The previous model for ZAP’s discrimination suggests specific recognition of viral transcripts by the zinc finger domain [14]. However, it is unclear whether such a model would suffice to explain its anti-retroelement restriction. For instance, ZAP inhibits not just human LINE-1 but also other mobile elements, including codon-optimized human and mouse LINE-1s, a zebrafish LINE-2, a mouse endogenous retrovirus, and the non-autonomous SINE element Alu [6,7]. This breadth of binding suggests ZAP’s specificity is unlikely to derive from recognition of a specific RNA sequence or structure. One potentially intriguing clue emerges from ZAP’s degradation of full-length but not truncated LINE-1 transcripts. If these shorter transcripts are not properly capped or polyadenylated, or have been decapped in preparation for degradation, one could propose that ZAP recognizes some post-transcriptional modification of RNAs to achieve specificity.

It is also possible that ZAP does not always discriminate between self and non-self. Indeed, recent findings suggest that ZAP also binds and regulates a subset of cellular mRNAs in the absence of infection or presumed LINE-1 retrotransposition [20]. It is tempting to speculate which came first. Possibly, ZAP was born to restrict endogenous elements and later co-opted for restriction of infectious viruses. It is also possible that ZAP’s ability to regulate self mRNAs preceded co-option for host defense. Alternatively, ZAP’s original role in host defense may have led to its subsequent co-option in the regulation of some specific mRNAs. These additional host RNA targets may well hold the key to uncovering ZAP’s specificity and subsequently understanding why it may be efficient to evolve host factors that restrict both endogenous elements and infectious viruses.
